# Comprehensive Analysis of Codon Usage Bias in Seven *Epichloë* Species and Their Peramine-Coding Genes

**DOI:** 10.3389/fmicb.2017.01419

**Published:** 2017-07-27

**Authors:** Hui Song, Jing Liu, Qiuyan Song, Qingping Zhang, Pei Tian, Zhibiao Nan

**Affiliations:** State Key Laboratory of Grassland Agro-ecosystems, College of Pastoral Agriculture Science and Technology, Lanzhou University Lanzhou, China

**Keywords:** codon usage, *Epichloë* species, mutation pressure, natural selection, peramine, selection pressure

## Abstract

Codon usage bias plays an important role in shaping genomes and genes in unicellular species and multicellular species. Here, we first analyzed codon usage bias in seven *Epichloë* species and their peramine-coding genes. Our results showed that both natural selection and mutation pressure played a role in forming codon usage bias in seven *Epichloë* species. All seven *Epichloë* species contained a peramine-coding gene cluster. Interestingly, codon usage bias of peramine-coding genes were not affected by natural selection or mutation pressure. There were 13 codons more frequently found in *Epichloë* genome sequences, peramine-coding gene clusters and orthologous peramine-coding genes, all of which had a bias to end with a C nucleotide. In the seven genomes analyzed, codon usage was biased in highly expressed coding sequences (CDSs) with shorter length and higher GC content. Genes in the peramine-coding gene cluster had higher GC content at the third nucleotide position of the codon, and highly expressed genes had higher GC content at the second position. In orthologous peramine-coding CDSs, high expression level was not significantly correlated with CDS length and GC content. Analysis of selection pressure identified that the genes orthologous to peramine genes were under purifying selection. There were no differences in codon usage bias and selection pressure between peramine product genes and non-functional peramine product genes. Our results provide insights into understanding codon evolution in *Epichloë* species.

## Introduction

The genetic code constitutes of 64 triplet codons encoding for 20 amino acids, with synonymous codons coding for the same amino acid. Synonymous codons occur at different frequencies in genomes/genes, a phenomenon referred as codon usage bias (Hershberg and Petrov, 2008; Plotkin, 2011). Mutational pressure and natural selection are considered to be the two major factors contributing to codon usage bias (Hershberg and Petrov, 2008). Early studies into codon usage bias focused on the connection between mutational pressure and natural selection based on the AT/GC content in prokaryotes. Fox example, mutation pressure was shown to be the major force shaping codon usage in *Rickettsia prowazekii* and *Borrelia burgdorferi*, both of which have high AT content (Andersson et al., 1998; McInerney, 1998). In contrast, *Mycobacterium tuberculosis* has high GC content, and analysis of the genome suggested that codon usage bias experienced selection pressure in this species (de Miranda et al., 2000). Increasing number of species suggest that codon usage in prokaryotes and eukaryotes may result from an equilibrium between mutation and selection pressures (Hershberg and Petrov, 2008). In an analysis of 100 eubacterial and archaeal genomes, authors found that genome-wide codon usage bias was primarily driven by mutational pressure that acts throughout the genome, and secondarily by selective forces acting on translated sequences (Chen et al., 2004). In *Aspergillus*, mutation pressure influences codon usage bias in low-expression genes, and selection driven codon usage bias in high-expression genes (Lloyd and Sharp, 1991; Iriarte et al., 2012). In addition, codon usage bias plays an important role in gene expression. Zhou et al. (2016) demonstrated that codons in *Neurospora* preferentially used toward ending with G or C nucleotides, but that codon usage contributed to differences in gene expression though its effects on transcription. Codon usage bias can influence translation speed, and often plays a role in the evolution of highly expressed genes, such as *tuf* genes in *Salmonella* (Brandis and Hughes, 2016). Therefore, studying codon usage bias and evolutionary forces that shape codon usage bias is important for our understanding of how genomes evolve.

The sexual and asexual states of endophytic fungi belonging to the genus *Epichloë* have been identified in cool season grass (Poaceae) worldwide. To date, 43 *Epichloë* endophytes have been named (Leuchtmann et al., 2014), and molecular evidence suggests that these *Epichloë* species were derived in Eurasia (Song and Nan, 2015; Song et al., 2016a). *Epichloë* species produce bioactive alkaloids that can protect to the host plant (Schardl et al., 2012, 2013b; Song et al., 2016a). These alkaloids include the indole-diterpene lolitrem B, ergot alkaloids, lolines, and peramine (Schardl et al., 2012, 2013a). While the alkaloids can be beneficial for grass, indole-diterpene lolitrem B and ergot alkaloid ergovaline harm the health of livestock that graze on infected pastures (Schardl et al., 2012, 2013b). Lolines and peramine can protect host plants from feeding by insects (Schardl et al., 2012, 2013b). The ecology and physiology of *Epichloë* endophytes are relatively well-understood, but few studies have investigated the evolution of *Epichloë* species using molecular methods (Song et al., 2016a). Here, we identified alkaloids-coding genes and analyzed codon usage bias in seven asexual *Epichloë* species and their alkaloids-coding genes with available coding sequences (CDSs) data (Schardl et al., 2013a, 2014; Pan, 2014; Chen et al., 2015): *Epichloë amarillans* E4668, *Epichloë bromicola* AL0434, *Epichloë festucae* E894, *Epichloë glyceriae* E277, *Epichloë sylvatica* E7368, *Epichloë typhina* E8 and *Epichloë typhina* subsp. *poae* E5819. We found peramin-coding gene clusters in all seven genomes. Furthermore, we analyzed codon usage bias of the peramin-coding gene cluster, and compared gene-specific codon usage bias to genomic codon usage bias. These results provide new insights into understanding the molecular evolution of *Epichloë* species.

## Materials and methods

### Sequence retrieval

The CDSs of seven *Epichloë* species were obtained from genome projects at University of Kentucky (www.endophyte.uky.edu/) (Schardl et al., 2013a, 2014; Pan, 2014; Chen et al., 2015). The *Epichloë* species that were used in this study were *Epichloë amarillans* E4668, *Epichloë bromicola* AL0434, *Epichloë festucae* E894, *Epichloë glyceriae* E277, *Epichloë sylvatica* E7368, *Epichloë typhina* E8, and *Epichloë typhina* subsp. *poae* E5819 (Table 1). The following evaluation criteria were adopted to avoid bias against on short and partial sequences (Song et al., 2016b): (1) CDS length of 300 bp or more; (2) CDS starting in ATG and ending in TAA, TAG or TGA and (3) CDS lacking premature termination or ambiguous codons.

**Table 1:** The seven *Epichloë* species in this study.

**Organism**	**Lab ID**	**Host**	**Total CDSs in genome**	**Total CDSs in this study**
*Epichloë amarillans*	E4668	*Agrostis hyemalis*	12,283	8,210
*Epichloë bromicola*	AL0434	*Bromus tomentellus*	11,669	8,202
*Epichloë festucae*	E894	*Festuca trachyphylla*	10,502	8,271
*Epichloë glyceriae*	E277	*Glyceria striata*	11,761	10,059
*Epichloë sylvatica*	E7368	*Brachypodium sylvaticum*	17,587	7,737
*Epichloë typhina*	E8	*Lolium perenne*	11,965	8,523
*Epichloë typhina* subsp. *poae*	E5819	*Poa nemoralis*	9,079	7,854

### Calculation of codon index

Codon W (version 1.4, http://codonw.sourceforge.net) was used to calculate the codon adaptation index (CAI), effective number of codon (ENC), relative synonymous codon usage (RSCU), and CDS length. GC content at the first (GC1), second (GC2), and third (GC3) codon positions were calculated using an in-house Perl script (Additional File 1).

CAI values are between 0 and 1, where values closer to 1 suggest that a gene has experienced stronger selection to maintain a specific codon usage bias that is optimized for efficient translation (Sharp and Li, 1987). CAI can also serve as a proxy for gene expression levels (Sharp and Li, 1987; Vishnoi et al., 2010). The CAI values approaching 1 indicate that the gene is highly expressed. ENC is a non-directional measure that is dependent upon the nucleotide composition of genes. ENC values start from 20, indicating one codon was exclusively used to code for a given amino acid, and can be up to 61, indicating all codons were used equally (Wright, 1990). RSCU values larger than 1 indicate that there is a higher frequency of a particular codon in the genome than expected, while RSCU values <1 indicate that a codon is less frequent within the genome (Sharp and Li, 1987).

### Identification of alkaloid-coding genes

Gene families contain gene clusters that are a set of homologous genes within one organism. A gene cluster is a group genes found within the genome that encode for similar proteins, which share a generalized function and are often located within a few thousand base pairs of each other. Alkaloid-coding genes in *Epichloë* are often found in a gene cluster containing 10–11 genes (Schardl et al., 2012, 2013a). We used CDSs cluster of indole-diterpene lolitrem B from *E. festucae* (GenBank: JN61338, JN61339, and JN613320), ergot alkaloids from *Epichloë coenophiala* (GenBank: KC989569 and KC989570), lolines from *E. festucae* (GenBank: EF012267 and FJ594413), and peramine from *E. festucae* (GenBank: AB205145) as query to search for homologous genes in seven *Epichloë* genomic CDSs using local BLASTN program (Altschul et al., 1997). The following evaluation criteria were used as thresholds to determine inclusion in the subsequent analysis: (1) length of aligned sequences > 80%, (2) identity > 96% and (3) *E*-value ≤ 10^−10^. The matching alkaloid-coding sequences were extracted using an in-house Perl script (Additional File 2).

### Determining selection pressure

MAFFT (Katoh and Standley, 2013) was used to alignment orthologous gene pairs. PAL2NAL program (Suyama et al., 2006) was used to convert protein sequences into corresponding nucleotide sequences. PAML 4.0 (Yang, 2007) was used to calculate the K_a_/K_s_ (non-synonymous/synonymous per site substitution rates) ratio. Generally, K_a_/K_s_ = 1, >1, and <1 indicated neutral, positive, and purifying selection, respectively.

### Correlation analysis

We constructed linear regression tests that incorporated various measurements for codon usage bias as predictor parameters to estimate regression coefficients. The parameters included ENC, CAI, CDS length, GC1 content, GC2 content, GC3 content, and overall GC content. Correlation analyses were conducted in JMP 9.0 (SAS Institute, Inc., Cary, NC, USA). The student *t*-test was performed, and *P*-values of < 0.05 were considered significant.

## Results

### Base composition of seven *Epichloë* genomes

**Figure 1:**
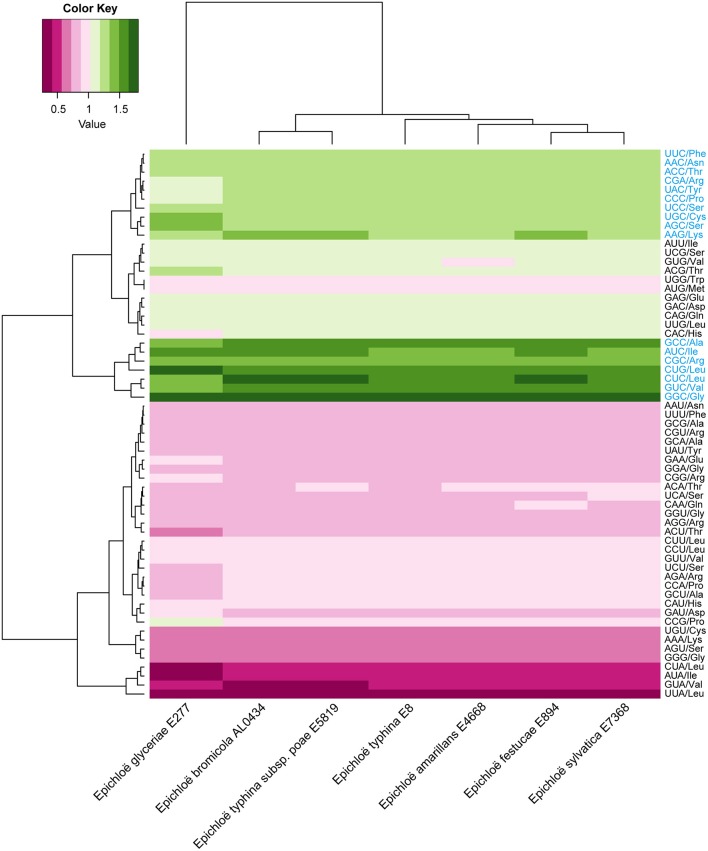
Codon usage frequency based on RSCU values in seven *Epichloë* genomes. The RSCU value was generated using codon W. The figure was generated using R script. More frequently used codons are indicated in blue font.

A total of 8,210 *E. amarillans* E4668, 8,202 *E. bromicola* AL0434, 8,271 *E. festucae* E894, 10,059 *E. glyceriae* E277, 7,737 *E. sylvatica* E7368, 8,523 *E. typhina* E8, and 7,854 *E. typhina* subsp. *poae* E5819 CDSs were used in this study based on our screening criteria (see Materials and Methods, Table 1). GC content at the three positions varied, and we found that the average GC content at the third position (GC3) was larger than the average GC content at the second position (GC2). The lowest was average GC content at the first position (GC1, Table 2). The average GC content at all three positions was higher than 50%, indicating that *Epichloë* had higher GC content than average AT content in CDSs. We found that the RSCU value of each codon was similar in across the seven *Epichloë* genomes that were analyzed. Seventeen codons had RSCU values higher than 1, and these codons were biased toward ending with G or C nucleotides (Figure 1). Furthermore, GGC (encoding Gly) had the highest RSCU value, and UUA (encoding Leu) had the lowest RSCU value, suggesting that GGC is used most frequently found codon in the *Epichloë* genomes, and UUA is the least frequent.

**Table 2:** GC content at three nucleotide positions of codons in seven *Epichloë* genomes.

**Organism**	**GC1 content**	**GC2 content**	**GC3 content**	**Overall GC content**
*Epichloë amarillans*	56.95	45.35	60.81	54.37
*Epichloë bromicola*	57.54	45.42	62.05	55.00
*Epichloë festucae*	57.41	45.36	61.44	54.74
*Epichloë glyceriae*	57.74	43.99	62.04	54.59
*Epichloë sylvatica*	57.55	45.41	61.84	54.93
*Epichloë typhina*	56.60	44.97	60.81	54.13
*Epichloë typhina* subsp. *poae*	57.95	45.52	62.51	55.33

**Figure 2:**
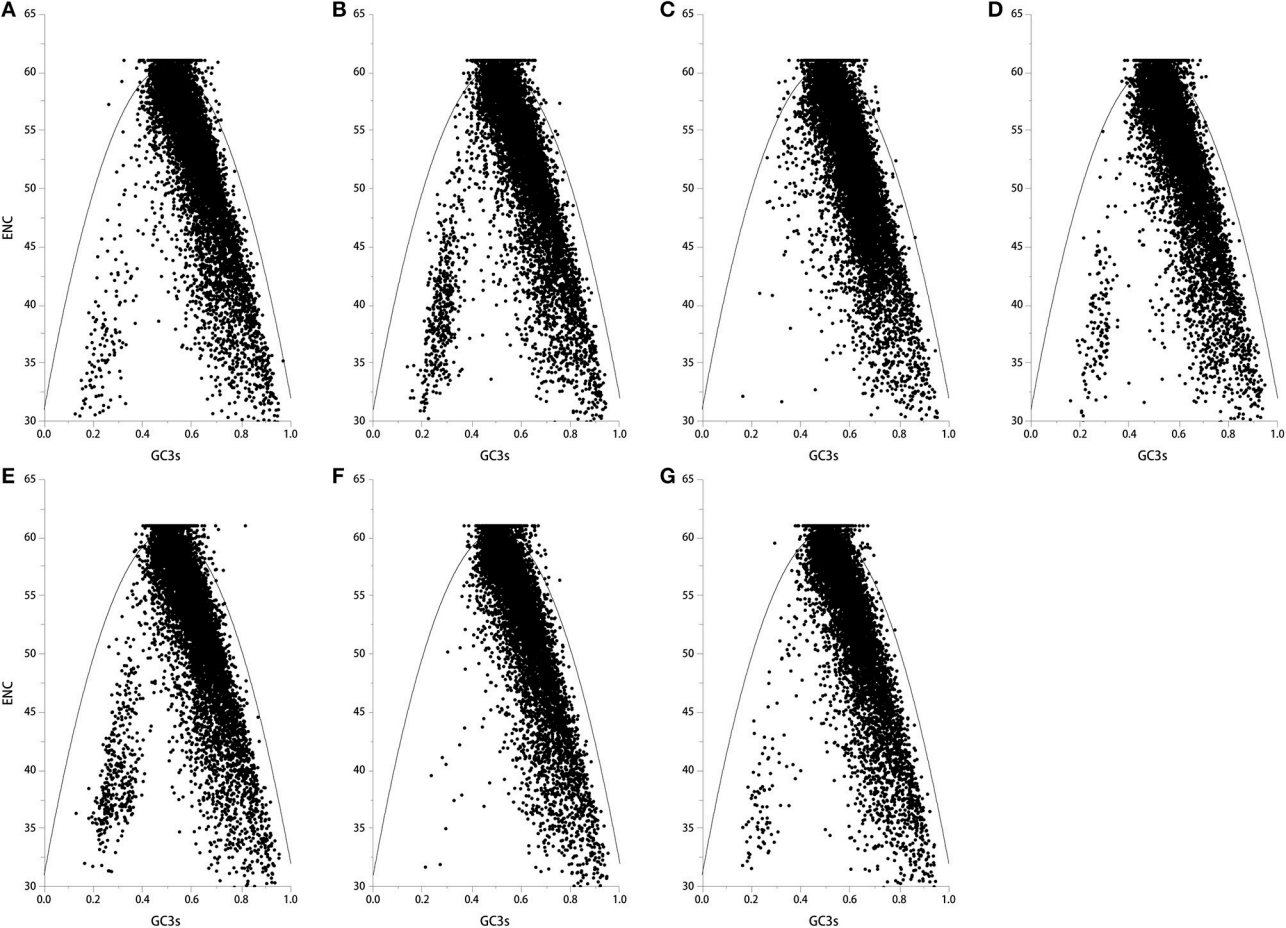
The ENC plot of the seven *Epichloë* genomes. The ENC value was generated using codon W. The figure was generated using Origin 9.0. The continuous curve indicates the relationship between ENC and GC3s values under neutral selection. The dot indicates a gene. **(A)** *Epichloë bromicola* AL0434, **(B)** *Epichloë typhina* E8, **(C)** *Epichloë glyceriae* E277, **(D)** *Epichloë festucae* E894, **(E)** *Epichloë amarillans* E4668, **(F)** *Epichloë typhina* subsp. *poae* E5819, **(G)** *Epichloë sylvatica* E7368.

If codons are constrained by neutral selection pressure, genes can be located on one curve line in the ENC-plot (a plot of ENC vs. GC3s) (Wright, 1990). Genes that are all below or above the ENC curve are likely under positive or negative selection pressure for codon usage. Kawabe and Miyashita (2003) demonstrated that if GC content in synonymous codon (GC3s) values across genes are narrow or broad, natural selection or mutation pressure may shape codon usage, respectively. Here, we found that most genes in the seven genomes fell below the ENC curve, where GC3s values were distributed in a broad range (*E. amarillans* E4668, *E. bromicola* AL0434, *E. glyceriae* E277, *E. sylvatica* E7368, and *E. typhina* subsp. *poae* E5819: 0.2–0.9; *E. festucae* E894, and *E. typhina* E8: 0.4–0.9, Figure 2), suggesting that mutation pressure is influencing codon usage patterns in these seven *Epichloë* genomes.

**Figure 3:**
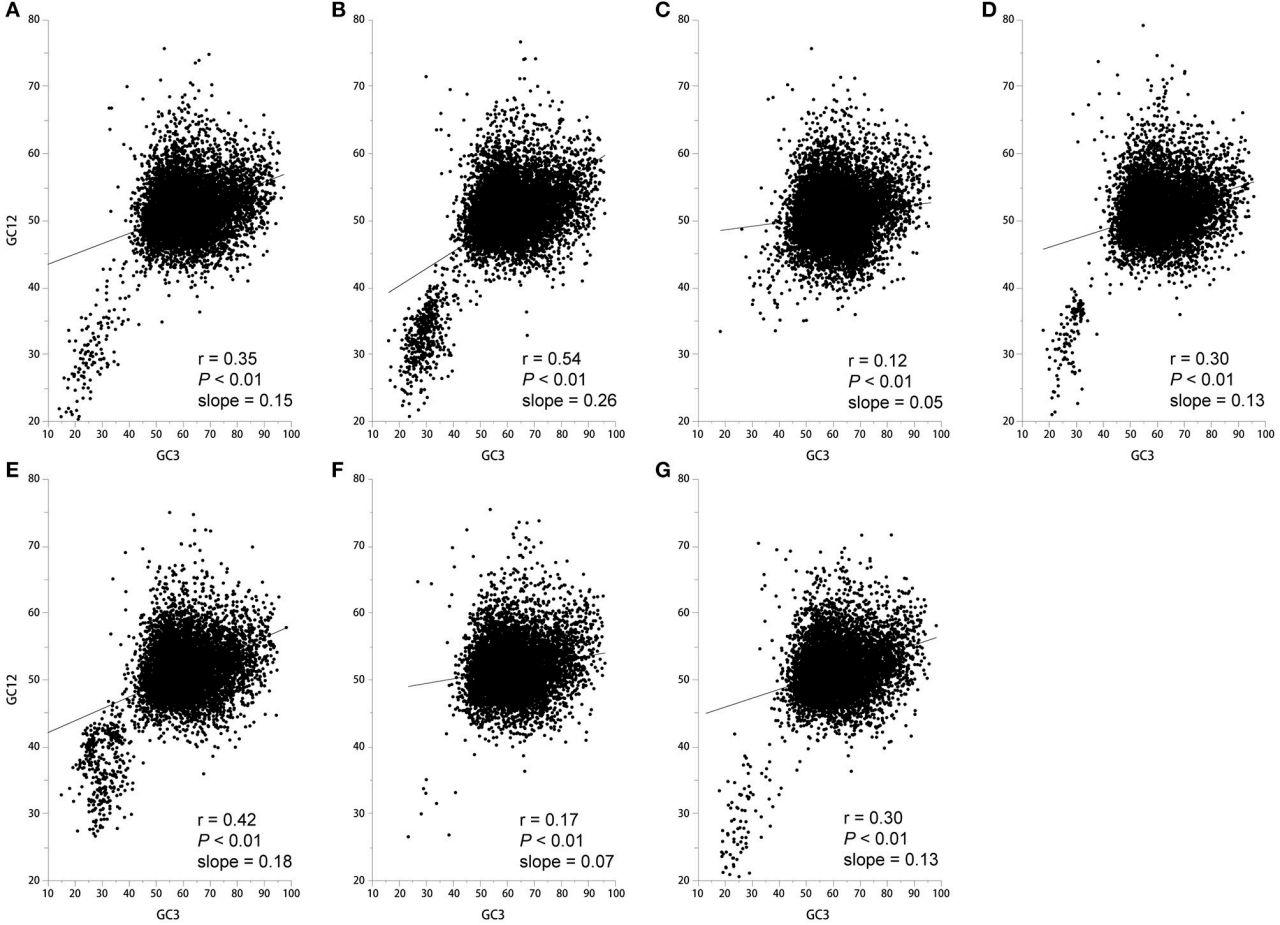
Correlation between GC12 and GC3 in the seven *Epichloë* genomes. GC content at the first (GC1), second (GC2), and third (GC3) codon positions were calculated using an in-house Perl script. Correlation analyses were executed in JMP 9.0, and the figure was generated using Origin 9.0. **(A)** *Epichloë bromicola* AL0434, **(B)** *Epichloë typhina* E8, **(C)** *Epichloë glyceriae* E277, **(D)** *Epichloë festucae* E894, **(E)** *Epichloë amarillans* E4668, **(F)** *Epichloë typhina* subsp. *poae* E5819, **(G)** *Epichloë sylvatica* E7368.

The neutrality plots that show a significant correlation between GC12 (average of GC1 and GC2 content) and GC3 with a slope approaching 0 suggest that natural selection is shaping codon usage (Sueoka, 1988). In contrast, a slope approaching to 1 suggests that mutation pressure is the dominant selection pressure (Sueoka, 1988). We found a significant positive correlation between GC12 and GC3 with a slope approaching 0 (Figure 3), therefore it is more likely that natural selection plays a role in shaping the codon usage pattern. Taken together, codon usage patterns of seven *Epichloë* genomes appear to be subject to both natural selection and mutation pressure.

### Correlation analysis of codon usage pattern in seven *Epichloë* genomes

We found a significant negative correlation between ENC and CAI in the *Epichloë* genomes (Table 3), indicating codon usage bias exists in highly expressed genes. In addition, the ENC value was positively correlated with CDS length (*P* < 0.01), but negatively correlated with GC3 content (*P* < 0.01), and overall GC content (*P* < 0.01, Table 3). However, the correlation among ENC value and both GC1 and GC2 was inconsistent. These results showed that codon usage bias was more prevelant in longer CDSs with higher GC3 and overall GC contents. However, GC1 and GC2 contents did not affect codon usage bias. CAI was positively correlated with GC3 content (*P* < 0.01), but inconsistently correlated with CDS length, GC1 content, GC2 content and overall GC content (Table 4). Taken together, GC3 content appears to affect gene expression, and higher GC3 content may increase gene expression levels in *Epichloë*.

**Table 3:** Correlation analysis between ENC and coding sequence architecture features in seven *Epichloë* genomes.

**ENC of strains**	**CAI**	**CDS length**	**GC1 content**	**GC2 content**	**GC3 content**	**Overall GC content**
*Epichloë amarillans*	−0.29^**^	0.25^**^	0.08^**^	0.08^**^	−0.40^**^	−0.20^**^
*Epichloë bromicola*	−0.32^**^	0.21^**^	−0.04^**^	0.05^**^	−0.61^**^	−0.39^**^
*Epichloë festucae*	−0.39^**^	0.21^**^	−0.04^**^	0.05^**^	−0.60^**^	−0.40^**^
*Epichloë glyceriae*	−0.51^**^	0.13^**^	−0.21^**^	0.07^**^	−0.76^**^	−0.58^**^
*Epichloë sylvatica*	−0.37^**^	0.22^**^	−0.09^**^	0.03^**^	−0.67^**^	−0.46^**^
*Epichloë typhina*	−0.17^**^	0.25^**^	0.15^**^	0.14^**^	−0.36^**^	−0.11^**^
*Epichloë typhina* subsp. *poae*	−0.45^**^	0.21^**^	−0.23^**^	−0.04^**^	−0.81^**^	−0.68^**^

** *Indicates significance at P < 0.01*.

**Table 4:** Correlation analysis between CAI and coding sequence architecture features in seven *Epichloë* genomes.

**CAI of strains**	**CDS length**	**GC1 content**	**GC2 content**	**GC3 content**	**Overall GC content**
*Epichloë amarillans*	0.03^*^	0.23^**^	−0.10^**^	0.40^**^	0.09^**^
*Epichloë bromicola*	0.005	0.19^**^	−0.13^**^	0.36^**^	0.25^**^
*Epichloë festucae*	−0.005	0.12^**^	−0.16^**^	0.35^**^	−0.03^*^
*Epichloë glyceriae*	0.005	−0.01	−0.35^**^	0.27^**^	0.02
*Epichloë sylvatica*	−0.01	0.12	−0.16^**^	0.35^**^	−0.03^*^
*Epichloë typhina*	0.06^**^	0.37^**^	0.05^**^	0.49^**^	0.43^**^
*Epichloë typhina* subsp. *poae*	−0.05^**^	−0.03^*^	−0.25^**^	0.28^**^	−0.20^**^

* *Indicates significance at P < 0.05*.

** *Indicates significance at P < 0.01*.

### Codon usage bias of peramine-coding gene clusters in *Epichloë* species

**Figure 4:**
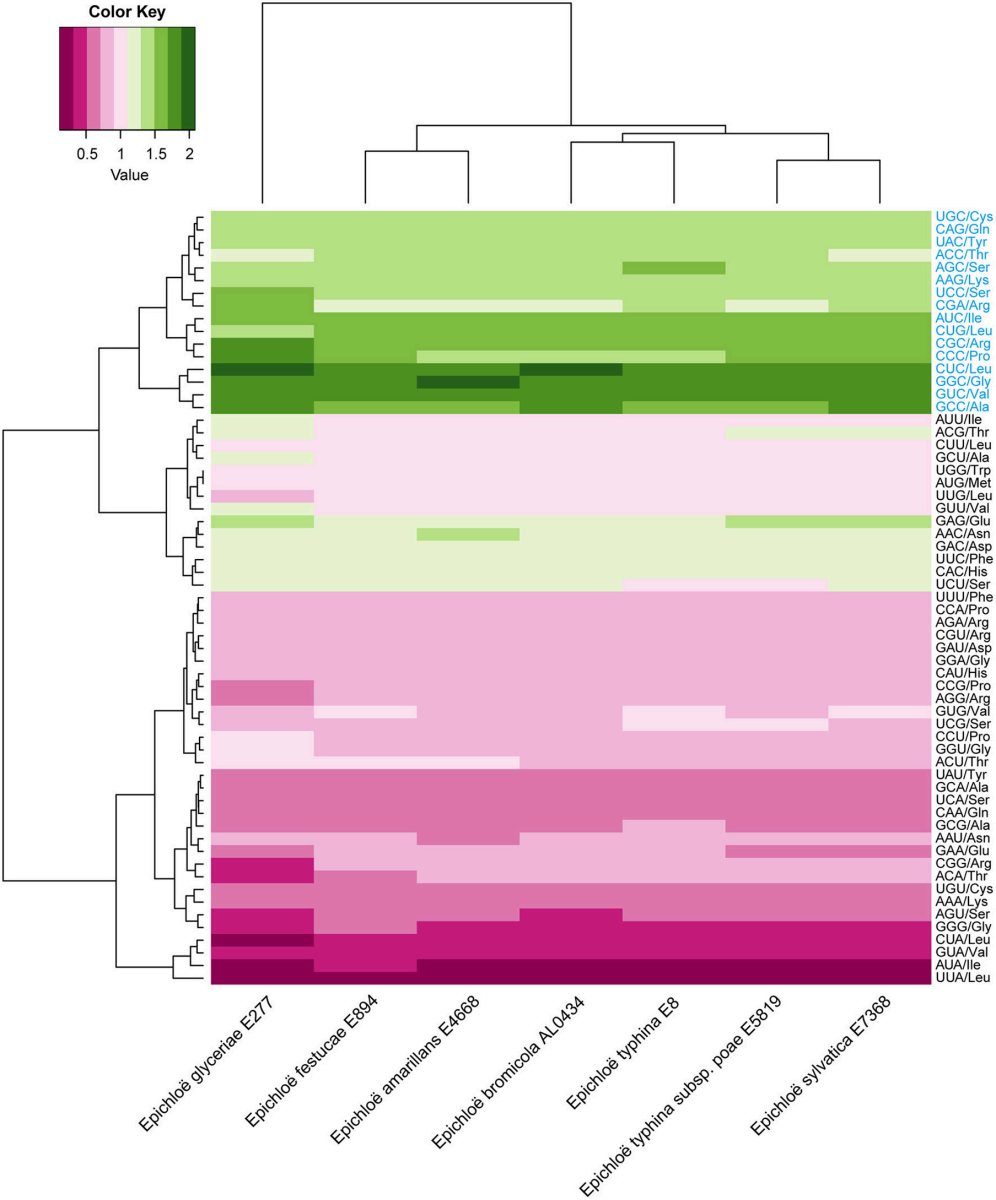
Codon usage frequency based on RSCU values in peramine-coding sequences. The RSCU value was generated using codon W. The figure was generated using R script. More frequently used codons are indicated in blue font.

Alkaloids produced in *Epichloë* species can increase host fitness and harm stock animals (Schardl et al., 2012, 2013b; Song et al., 2016a). Here, we investigated the evolution and gene expression of alkaloid–coding genes based on their codon usage pattern. We identified alkaloid-coding genes in the seven genomes by searching for homologous sequences of alkaloid genes that have already been identified in *Epichloë* species. We found peramine-coding gene clusters in all seven *Epichloë* species, and there were some losses of other alkaloid-coding gene clusters in the genomes as well (Table S1). The peramine-coding gene cluster contained 10 genes, including *EF100, EF101, EF102, perA, EF104, EF105, EF106, EF107, EF108*, and *EF109*. GC content at the three coding positions was similar within the peramine-coding gene cluster among the seven *Epichloë* species, following the GC3 > GC1 > GC2 pattern (Table S2). The average GC content was about 56% in each peramine-coding gene cluster, therefore GC content was higher than AT content in peramine-coding sequences, similar to the overall CDS-level GC/AT content in *Epichloë* species. We next calculated the RSCU values of each codon of peramine-coding genes, and found that the patterns were similar across the seven *Epichloë* genomes (Figure 4). Sixteen codons had RSCU values higher than 1, indicating that these 16 codons were more frequently used. GGC (encoding Gly) had the highest RSCU value, and UUA (encoding Leu) had the lowest RSCU value. The results suggested GGC as the most common codon in peramine-coding genes, and UUA was the least frequent. Furthermore, these 16 codons showed bias toward ending with G or C, with the exception of CGA (Figure 4).

In peramine-coding gene clusters, there was a positive, but not significant, correlation between GC12 and GC3 with a slope approaching 0 (Figure S1), suggesting that influences other than natural selection and mutation pressure played a role in shaping the codon usage pattern. ENC was negatively correlated with average GC3 and average overall GC content in peramine-coding gene clusters in the seven *Epichloë* genomes (Table 5). These results indicate that average GC3 and overall GC content both affected codon usage, and higher GC3 and overall GC contents could increase codon usage bias in *Epichloë* genomes in peramine-coding gene clusters. CAI was positively correlated with GC2 content (Table 6), therefore GC2 content may be affecting gene expression, and higher GC2 content could increase expression of peramine-coding genes.

**Table 5:** Correlation analysis between ENC and coding sequence architecture features in peramine-coding sequences.

**ENC of genes**	**CAI**	**CDS length**	**GC1 content**	**GC2 content**	**GC3 content**	**Overall GC content**
*Epichloë amarillans*	−0.15	0.27	0.002	0.03	−0.76^*^	−0.65^*^
*Epichloë bromicola*	−0.37	0.39	−0.25	0.40	−0.85^**^	−0.72^**^
*Epichloë festucae*	−0.49	0.41	−0.33	0.16	−0.86^**^	−0.73^*^
*Epichloë glyceriae*	−0.47	0.19	−0.40	0.30	−0.89^**^	−0.84^**^
*Epichloë sylvatica*	−0.33	0.33	−0.21	0.30	−0.83^**^	−0.72^*^
*Epichloë typhina*	−0.35	0.35	−0.30	0.34	−0.86^**^	−0.77^**^
*Epichloë typhina* subsp. *poae*	−0.33	0.37	−0.28	0.30	−0.86^**^	−0.77^**^

* *Indicates significance at P < 0.05*.

** *Indicates significance at P < 0.01*.

**Table 6:** Correlation analysis between CAI and coding sequence architecture features in peramine-coding sequences.

**CAI of genes**	**CDS length**	**GC1 content**	**GC2 content**	**GC3 content**	**Overall GC content**
*Epichloë amarillans*	−0.02	0.42	−0.83^**^	0.29	0.15
*Epichloë bromicola*	−0.09	0.59	−0.90^**^	0.30	0.22
*Epichloë festucae*	−0.09	0.54	−0.92^**^	0.32	0.21
*Epichloë glyceriae*	−0.03	0.65	−0.88^**^	0.32	0.27
*Epichloë sylvatica*	−0.08	0.53	−0.90^**^	0.35	0.24
*Epichloë typhina*	−0.09	0.55	−0.91^**^	0.32	0.22
*Epichloë typhina* subsp. *poae*	−0.09	0.59	−0.91^**^	0.36	0.27

** *Indicates significance at P < 0.01*.

### Codon usage bias of genes orthologous to peramine-coding genes in seven *Epichloë* species

**Figure 5:**
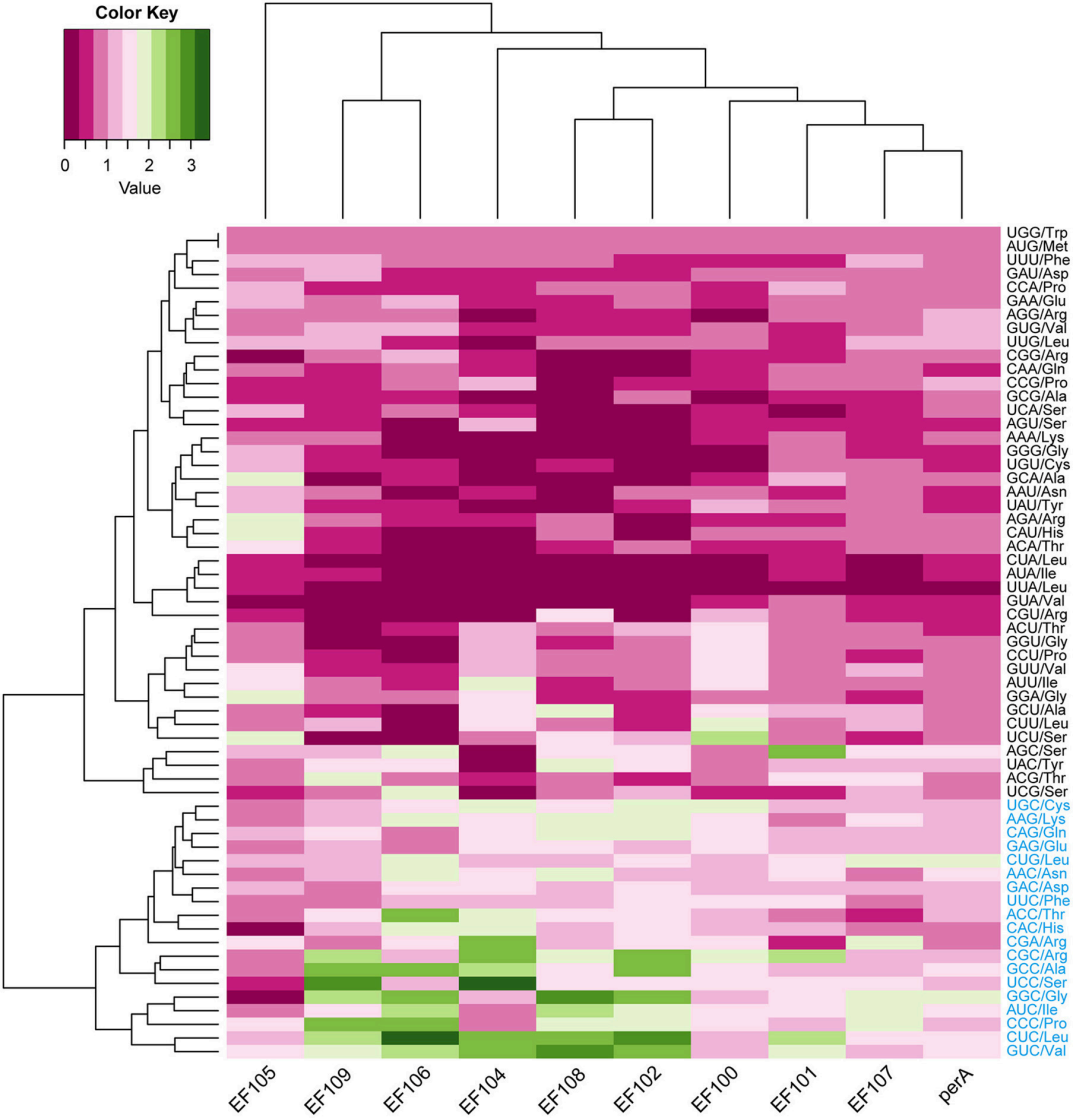
Codon usage frequency based on RSCU values in orthologous peramine-coding sequences. The RSCU value was generated using codon W. The figure was generated using R script. More frequently used codons are indicated in blue font.

Orthologous genes are distributed in different species that diverged from a single ancestral gene after a speciation event (Kuzniar et al., 2008). GC content at the three codon positions differed in orthologous peramine-coding genes among the seven *Epichloë* species, but the pattern was similar, presenting the GC3 > GC1 > GC2 pattern except for *EF100* and *EF105* (Table S3). The average GC content was higher than 50% in orthologous peramine-coding genes, indicating the average GC content was higher than AT content in orthologous peramine-coding genes. The exception to this pattern was observed in EF105, which had higher AT content over GC content. Nineteen codons had RSCU values larger higher than 1, indicating that these 19 codons were more frequently found in orthologous peramine-coding genes. Similar to the results from our analysis of the genome and peramine-coding gene clusters, these 19 codons were biased toward ending in G or C, except for CGA (Figure 5). Comparing the RSCU values from analysis of the *Epichloë* genomes, peramine-coding gene clusters and orthologous peramine-coding genes, we found 13 codons that were most frequently present in *Epichloë*, including UGC (encoding Cys), AAG (encoding Lys), CUG (encoding Leu), ACC (encoding Thr), CGA (encoding Arg), CGC (encoding Arg), GCC (encoding Ala), UCC (encoding Ser), GGC (encoding Gly), AUC (encoding Ile), CCC (encoding Pro), CUC (encoding Leu), and GUC (encoding Val). These 13 codons were biased toward ending in C.

**Figure 6:**
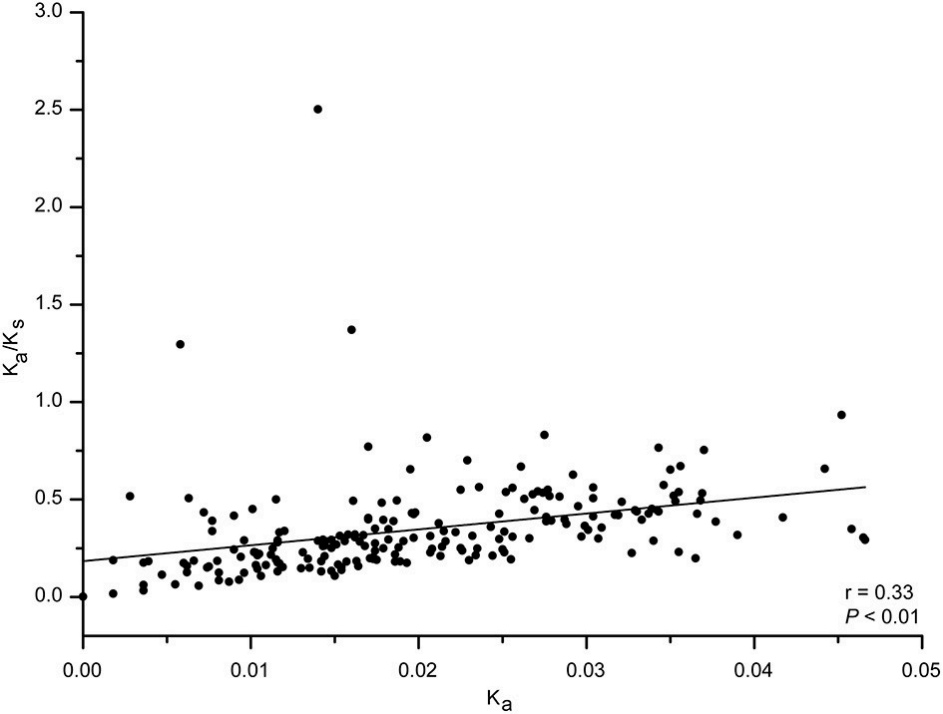
The K_a_/K_s_ value in orthologous peramine-coding sequences. PAL2NAL was used to convert amino acid sequences into the corresponding nucleotide sequences. PAML 4.0 was used to calculate the non-synonymous/synonymous substitution (K_a_/K_s_) ratio. K_a_/K_s_ values of 1, >1, and <1 indicated neutral, positive, and purifying selection, respectively. Correlation analyses were executed in JMP 9.0, and the figure was generated using Origin 9.0.

We next analyzed codon usage bias in orthologous peramine-coding genes. The slope of the relationship between GC12 and GC3 ranged from −1.04 to 0.37, and there were no significant correlations between GC12 and GC3 (Figure S2). This suggests that natural selection and mutation pressure did not play a major role in shaping codon usage bias. ENC was inconsistently correlated with CAI, CDS length, GC1, GC2, GC3, and overall GC (Table 7). We also observed inconsistent correlation between CAI and CDS length, GC1, GC2, GC3, and overall GC in orthologous peramine-coding genes (Table 8). The K_a_/K_s_ value was <1, indicating that these orthologous peramine-coding genes were subject to purifying selection (Figure 6). However, K_a_/K_s_ values from three orthologous gene pairs were larger than 1, therefore these genes likely underwent positive selection (Figure 6). In addition, the average K_a_/K_s_ value of *EF101* genes had the highest value, and *EF100* genes had the lowest value (Figure S3), indicating that the *EF100* genes are likely functionally conserved and *EF101* may be functionally derived compared to other orthologous gene pairs.

**Table 7:** Correlation analysis between ENC and coding sequence architecture features in orthologous peramine-coding sequences.

**ENC of genes**	**CAI**	**CDS length**	**GC1 content**	**GC2 content**	**GC3 content**	**Overall GC content**
EF100	0.03	−0.22	0.53	0.40	0.60	0.68
EF101	−0.78^*^	−0.16	0.05	−0.45	−0.20	−0.32
EF102	−0.35	0.48	−0.77^*^	0.06	−0.64	−0.61
perA	−0.29	−0.48	−0.68	−0.35	−0.55	−0.98^**^
EF104	−0.84^*^	0.87^*^	−0.67	0.60	−0.54	−0.54
EF105	0.04	−0.81^*^	−0.42	−0.61	0.06	−0.55
EF106	−0.98^**^	0.81^*^	−0.54	0.68	−0.92^**^	−0.84^*^
EF107	0.06	−0.11	0.06	−0.41	0.25	0.05
EF108	−0.11	−0.5	0.7	−0.34	−0.26	0
EF109	−0.82^*^	0	0.92^**^	0.85^*^	−0.71	0.22

* *Indicates significance at P < 0.05*.

** *Indicates significance at P < 0.01*.

**Table 8:** Correlation analysis between CAI and coding sequence architecture features in orthologous peramine-coding sequences.

**CAI of genes**	**CDS length**	**GC1 content**	**GC2 content**	**GC3 content**	**Overall GC content**
EF100	−0.92^**^	0.75	0.81^*^	−0.50	0.63
EF101	0.03	−0.39	0.53	0.63	0.27
EF102	0.21	0.33	0.15	−0.03	0.16
perA	0.28	0.31	0.07	0.24	0.37
EF104	−0.95^**^	0.89^**^	−0.67	0.82^*^	0.85^*^
EF105	−0.44	0.56	−0.58	0.12	−0.14
EF106	−0.79^*^	0.48	−0.62	0.94^**^	0.86^*^
EF107	−0.04	0.18	0.47	−0.55	−0.23
EF108	0.08	−0.49	−0.75	−0.03	−0.46
EF109	0	−0.92^**^	−0.81^*^	0.19	−0.71

* *Indicates significance at P < 0.05*.

** *Indicates significance at P < 0.01*.

## Discussion

A recent study on codon usage bias in *E. festucae* showed that both natural selection and mutation pressure played a role in forming codon usage bias in *E. festucae*, and that codon usage bias was influenced by CDS length (Li et al., 2016). There are 43 *Epichloë* species that have been reported to date, but it is not clear whether *Epichloë* species share similar codon usage bias. In this study, we conducted a comprehensive analysis of codon usage bias in seven *Epichloë* genomes and their peramine-coding genes. We found that the seven *Epichloë* genomes showed codon usage bias in CDSs with shorter length, and higher GC3 and overall GC content, and highly expressed genes had higher GC3 content. In the peramine-coding gene cluster, codon usage bias was higher in GC3 and overall GC content. In contrast to the CDS-wide analysis, highly expressed peramine-coding genes had higher GC2 content. In orthologous peramine-coding CDSs, there were no significant correlations between high expression level and CDS length or GC content.

The difference in codon usage bias between the *Epichloë* genome and peramine-coding gene clusters above mentioned may be considered as follows. Gene expression can be influenced by selection to optimize the translation of mRNA. Decreasing the pool of free ribosomes can decrease overall translational initiation rate, thereby lowering overall rate of protein production in *Salmonella* (Brandis and Hughes, 2016). Other factors that can influence codon bias include the levels of available tRNA, evolutionary pressures and rate of evolution of genes. In our analysis, we found that natural selection and mutational pressure both played an important role in forming codon usage bias in the *Epichloë* genomes. However, we did not find support that natural selection or mutation pressure influenced codon usage bias of peramine-coding genes. This suggests that codon usage bias in *Epichloë* genomes and peramine-coding genes may be under different pressures, highlighting the complexity of codon evolution.

Differences in GC3 content often influence gene expression levels (Hershberg and Petrov, 2008). However, we found that higher GC2 content was correlated with high expression levels in the peramine-coding gene cluster. To our knowledge, little is known about the role GC2 plays in gene expression patterns in fungi. Nevertheless, GC2 content plays a crucial role in influencing gene expression in cereal species (Poaceae) (Chakraborty and Paul, 2015). *Epichloë* endophytes broadly grow on cool-season grasses. The grass-*Epichloë* symbiosis provides the grass host protection from herbivorous insects by producing peramine in the form of secondary metabolites (Tanaka et al., 2005). Given this symbiotic relationship, the peramine-coding gene cluster may be under co-evolution with cool-season grasses.

*E. amarillans* E4668, *E. bromicola* AL0434, *E. festucae* E894, and *E. typhina* E8 strains produce peramine, but *E. glyceriae* E277, *E. sylvatica* E7368, and *E. typhina* subsp. *poae* E5819 strains cannot produce peramine (Schardl et al., 2012; Berry et al., 2015). *perA* gene is a key gene involved in the synthesis of peramine alkaloid (Berry et al., 2015). *E. glyceriae* E277 lost the *perA* gene (Table S1), and *E. sylvatica* E7368 and *E. typhina* subsp. *poae* E5819 contained a *perA*-ΔR^*^ allele, which results in a deletion of the C-terminal reductase domain in *perA*, rendering it non-functional (Berry et al., 2015). We did not find different codon usage bias and selection pressure in peramine product genes and non-functional peramine product genes.

In this study, we conducted a comprehensive analysis of codon bias bias in seven *Epichloë* genomes and their peramine-coding genes. We found that different evolutionary forces drive codon usage bias in genomic CDSs and peramine-coding genes. However, similar codon usage pattern and selection pressure were observed in peramine product genes and non-functional peramine product genes.

## Author contributions

HS and ZN conceived and designed research. HS analyzed data and wrote the manuscript. JL and QS analyzed data. QS, QZ, and PT participated in the discussion of the results. ZN contributed to the evaluation and discussion of the results and manuscript revision.

### Conflict of interest statement

The authors declare that the research was conducted in the absence of any commercial or financial relationships that could be construed as a potential conflict of interest. The reviewer VNK and handling Editor declared their shared affiliation, and the handling Editor states that the process nevertheless met the standards of a fair and objective review.

## Supplementary material

Figure S1 Correlation between GC12 and GC3 in peramine-coding sequences. GC content at the first (GC1), second (GC2), and third (GC3) codon positions were calculated using an in-house Perl script. Correlation analyses were executed in JMP 9.0, and the figure was generated using Origin 9.0. **(A)** *Epichloë bromicola* AL0434, **(B)** *Epichloë typhina* E8, **(C)** *Epichloë glyceriae* E277, **(D)** *Epichloë festucae* E894, **(E)** *Epichloë amarillans* E4668, **(F)** *Epichloë typhina* subsp. *poae* E5819, **(G)** *Epichloë sylvatica* E7368.

Figure S2 Correlation between GC12 and GC3 in orthologous peramine-coding sequences. GC content at the first (GC1), second (GC2), and third (GC3) codon positions were calculated using an in-house Perl script. Correlation analyses were executed in JMP 9.0, and the figure was generated using Origin 9.0. **(A)** EF100, **(B)** EF101, **(C)** EF102, **(D)** EF104, **(E)** EF105, **(F)** EF106, **(G)** EF107, **(H)** EF108, **(I)** EF109, **(J)** perA.

Figure S3 The average of K_a_/K_s_-value in orthologous peramine-coding sequences. PAL2NAL was used to convert amino acid sequences into the corresponding nucleotide sequences. PAML 4.0 was used to calculate the non-synonymous to synonymous per site substitution rates (K_a_/K_s_) ratio. K_a_/K_s_-values of 1, >1, and <1 indicated neutral, positive, and purifying selection, respectively. The figure was generated using Origin 9.0. **(A)** EF100, **(B)** EF101, **(C)** EF102, **(D)** perA, **(E)** EF104, **(F)** EF105, **(G)** EF106, **(H)** EF107, **(I)** EF108, **(J)** EF109.

Table S1 The information of alkaloid-coding sequences in the seven *Epichloë* genomes. ^+^Indicates that alkaloid-coding sequences were detected; ^−^Indicates that alkaloid-coding sequences were not detected.

Table S2 GC content at the three nucleotide positions in codons of peramine-coding sequences. GC content at the first (GC1), second (GC2), and third (GC3) codon positions were calculated using an in-house Perl script.

Table S3 GC content at the three nucleotide positions in codons of orthologous peramine-coding sequences. GC content at the first (GC1), second (GC2), and third (GC3) codon positions were calculated using an in-house Perl script.

Additional File 1 Perl script used to calculate GC content.

Additional File 2 Perl script used to extract alkaloid-coding sequences.
