# Supplementary figures and images for: Comprehensive Analysis of Codon Usage Bias in Seven Epichloë Species and Their Peramine-Coding Genes
Source: Front Microbiol. 2017 Jul 27;8:1419. doi: 10.3389/fmicb.2017.01419 (PMC5529348; doi:10.3389/fmicb.2017.01419)

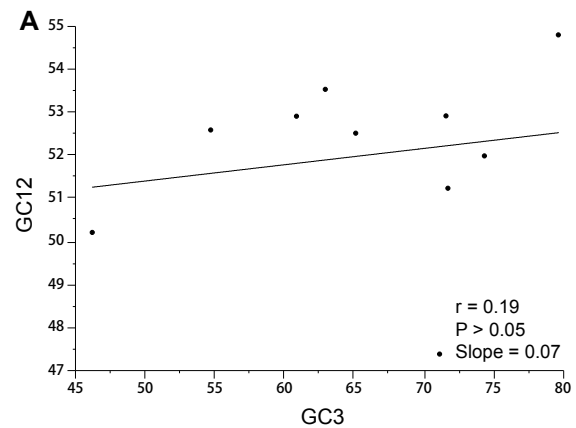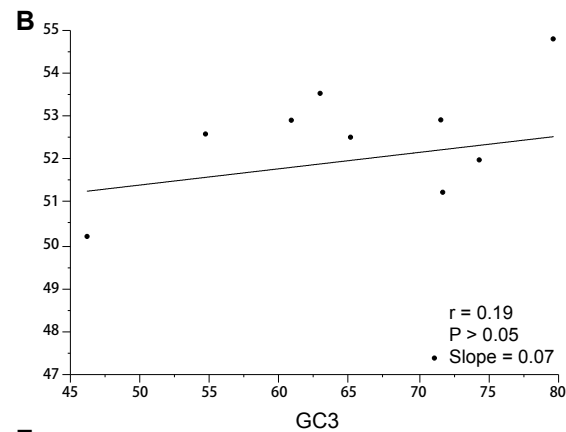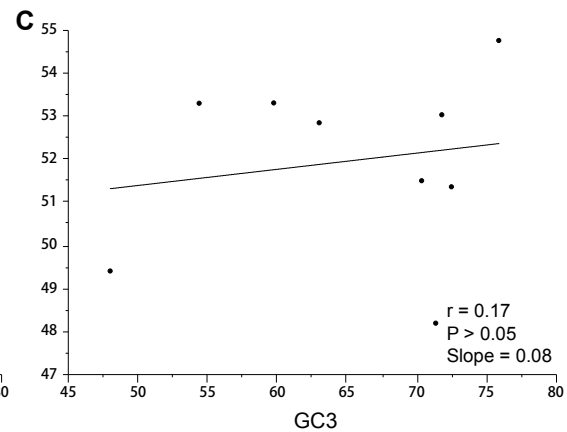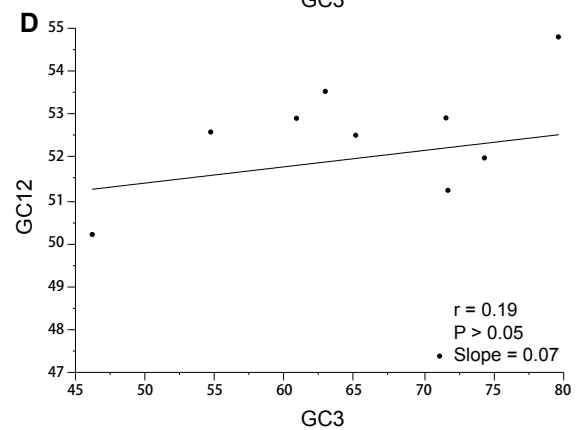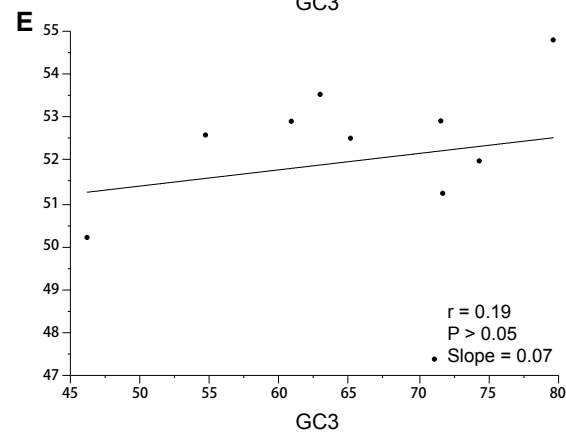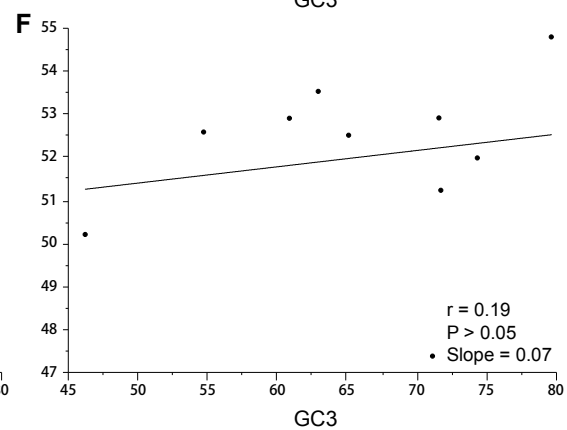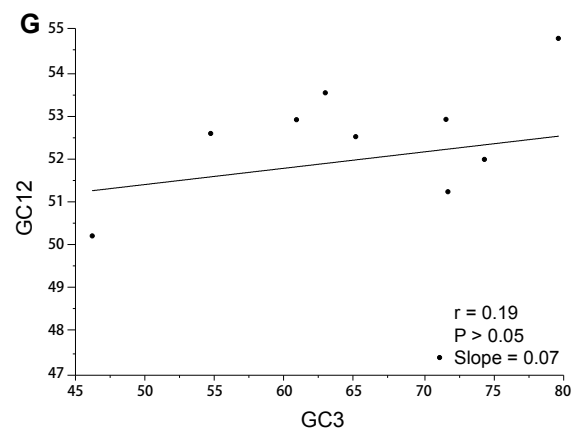

Supplement: Figure S1 — Correlation between GC12 and GC3 in peramine-coding sequences. GC content at the first (GC1), second (GC2), and third (GC3) codon positions were calculated using an in-house Perl script. Correlation analyses were executed in JMP 9.0, and the figure was generated using Origin 9.0. (A) Epichloë bromicola AL0434, (B) Epichloë typhina E8, (C) Epichloë glyceriae E277, (D) Epichloë festucae E894, (E) Epichloë amarillans E4668, (F) Epichloë typhina subsp. poae E5819, (G) Epichloë sylvatica E7368. [file Image1.PDF]

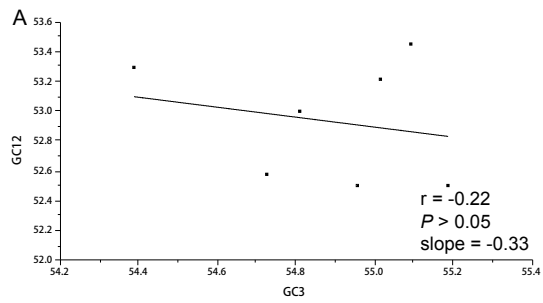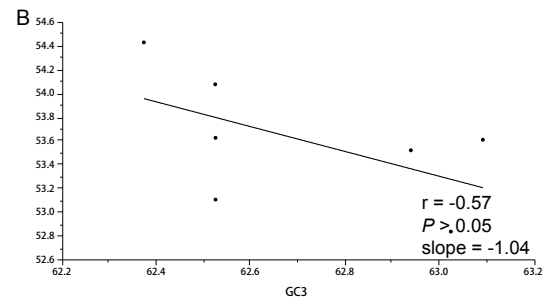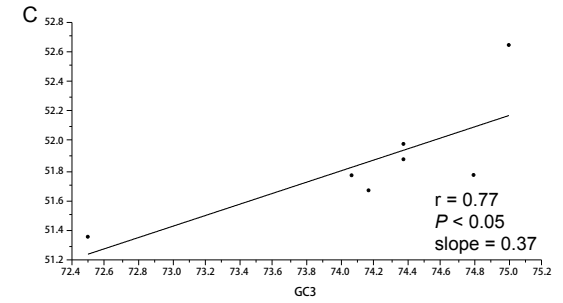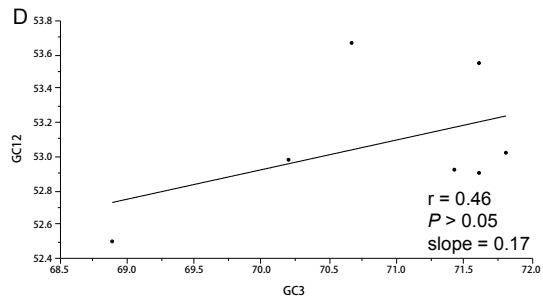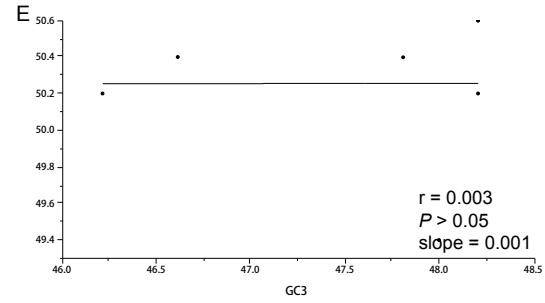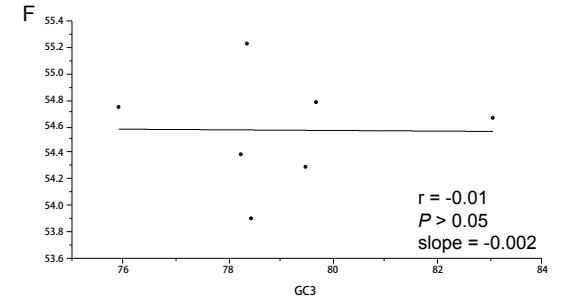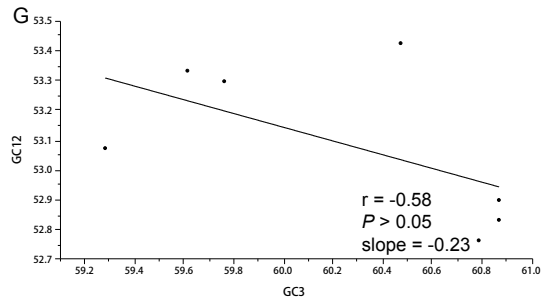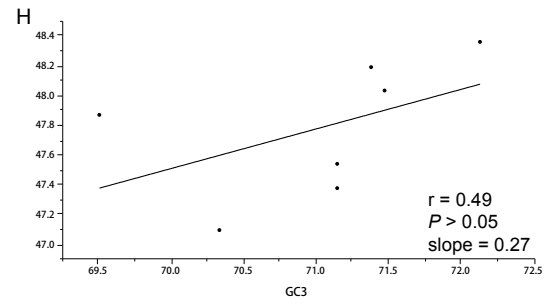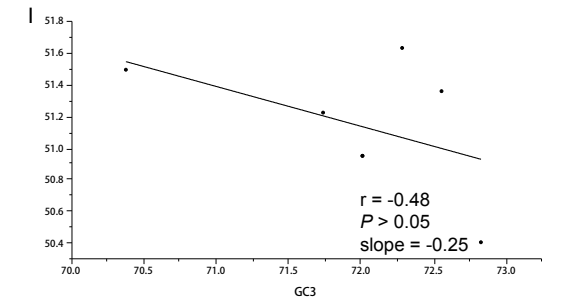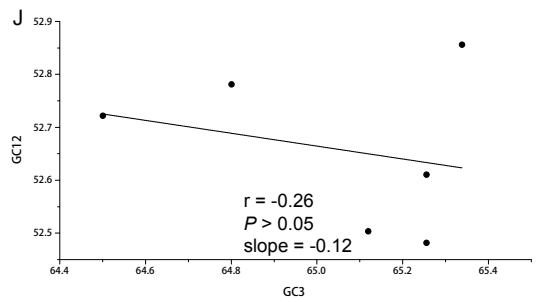

Supplement: Figure S2 — Correlation between GC12 and GC3 in orthologous peramine-coding sequences. GC content at the first (GC1), second (GC2), and third (GC3) codon positions were calculated using an in-house Perl script. Correlation analyses were executed in JMP 9.0, and the figure was generated using Origin 9.0. (A) EF100, (B) EF101, (C) EF102, (D) EF104, (E) EF105, (F) EF106, (G) EF107, (H) EF108, (I) EF109, (J) perA. [file Image2.PDF]

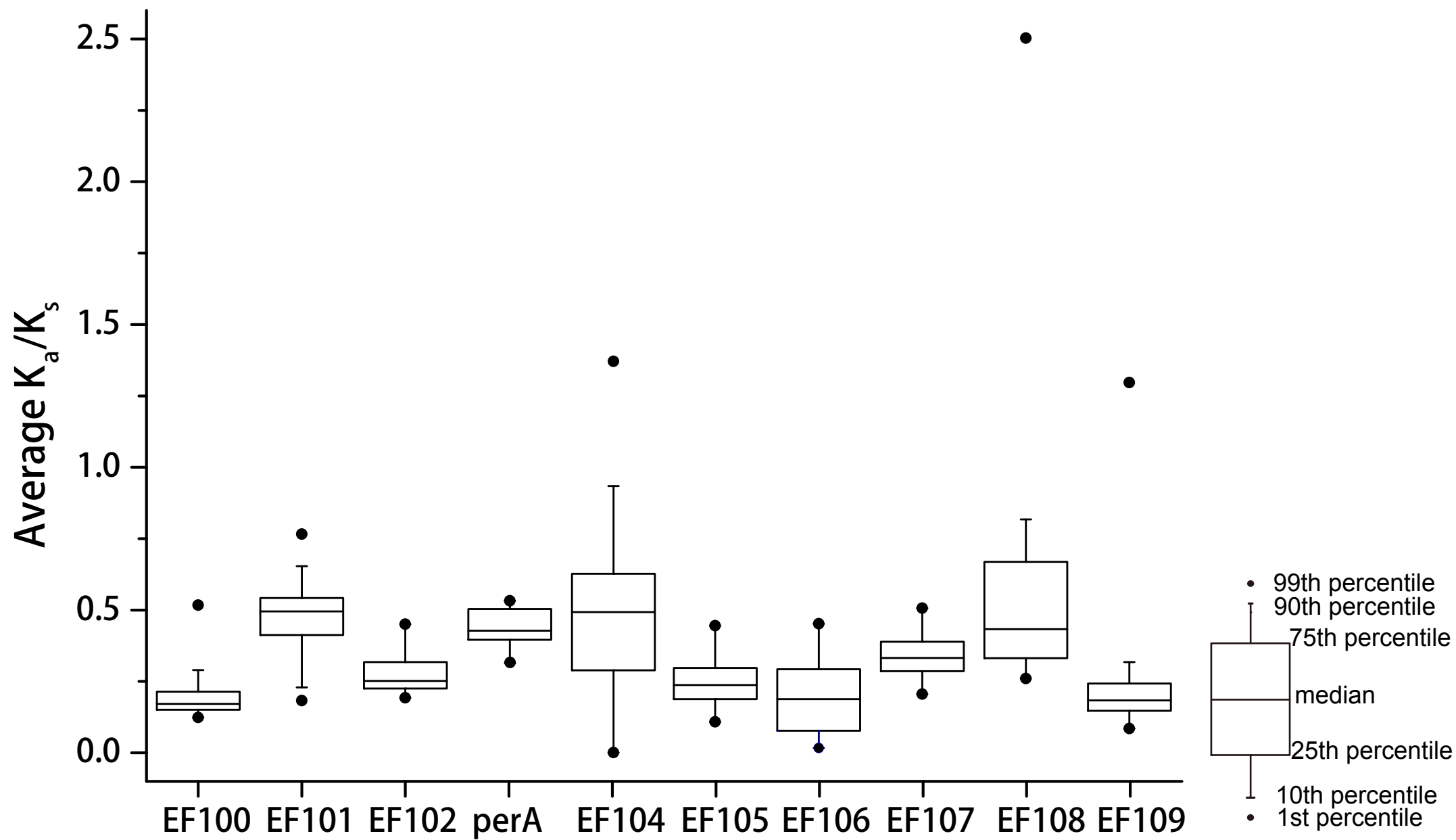

Supplement: Figure S3 — The average of Ka/Ks-value in orthologous peramine-coding sequences. PAL2NAL was used to convert amino acid sequences into the corresponding nucleotide sequences. PAML 4.0 was used to calculate the non-synonymous to synonymous per site substitution rates (Ka/Ks) ratio. Ka/Ks-values of 1, >1, and <1 indicated neutral, positive, and purifying selection, respectively. The figure was generated using Origin 9.0. (A) EF100, (B) EF101, (C) EF102, (D) perA, (E) EF104, (F) EF105, (G) EF106, (H) EF107, (I) EF108, (J) EF109. [file Image3.PDF]
