# Supplementary material for: Comprehensive Analysis of Codon Usage Bias in Seven Epichloë Species and Their Peramine-Coding Genes
Source: Front Microbiol. 2017 Jul 27;8:1419. doi: 10.3389/fmicb.2017.01419 (PMC5529348; doi:10.3389/fmicb.2017.01419)
Supplement: Additional File 1 — Perl script used to calculate GC content. [file DataSheet1.DOCX]

#!/usr/bin/perl

sub gc {

my $x;

if($_[0] eq "G" or $_[0] eq "C" ){

$x=1;

}

$x;

}

my $input="Name.fasta"; # File name

my ($li,$id,$i,$len,$s,$tm);

open TMP, $input || die;

open MOUT, ">output1.txt" || die;

print MOUT "CDS_id\tGC1\tGC2\tGC3\n";

while($li=<TMP>){

if($li=~/\>(\w+)/) {

print MOUT "$1\t";

}

else {

chomp $li;

$li=~s/\s//g;

$len=length $li;

$i=0;

my (@t,@g);

while($i<$len) {

$s=substr($li,$i,1);

$y=$i%3;

$t[$y]++;

$g[$y]+=gc($s);

$i++;

}

$tm=$g[0]/$t[0]*100;

printf MOUT "%.3f\t",$tm;

$tm=$g[1]/$t[1]*100;

printf MOUT "%.3f\t",$tm;

$tm=$g[2]/$t[2]*100;

printf MOUT "%.3f\n",$tm;

}

}

close TMP;

close MOUT;
