# Supplementary material for: Comprehensive Analysis of Codon Usage Bias in Seven Epichloë Species and Their Peramine-Coding Genes
Source: Front Microbiol. 2017 Jul 27;8:1419. doi: 10.3389/fmicb.2017.01419 (PMC5529348; doi:10.3389/fmicb.2017.01419)
Supplement: Additional File 2 — Perl script used to extract alkaloid-coding sequences. [file DataSheet2.DOCX]

#!/usr/bin/perl -w

open(LIST,"list.txt")||die "$!";

while(my $_=<LIST> ){

chomp;

$_=~ s/\s+$//;

$_=~s/^\s+//;

$hash{$_}=1;

}

close(LIST);

open(IN,"oneline")||die "$!";

open(OUT,">result.fasta")||die "$!";

while($head=<IN> ){

$head=~s/>//;

$head=~ s/\s+$//;

$head=~s/^\s+//;

chomp;

$seq=<IN>;

chomp $seq;

$seq=~ s/\s+$//;

$seq=~s/^\s+//;

my ($name)=$head;

if(exists $hash{$name}){

print OUT ">$head\n$seq\n";

}

}

close(IN);

close(OUT);
